# Morphometric and histological changes in cardiac nodes after acute spontaneous myocardial infarction in humans and pigs

**DOI:** 10.14202/vetworld.2024.2880-2888

**Published:** 2024-12-19

**Authors:** Fabián Gómez-Torres, Luis Ernesto Ballesteros-Acuña, Pilar Molina-Aguilar, César Ríos-Navarro, Amparo Ruíz-Sauri

**Affiliations:** 1 Department of Basic Sciences, School of Medicine, Universidad Industrial de Santander, Cra 32 # 29-31, 68002, Bucaramanga, Colombia; 2 Department of Pathology, Faculty of Medicine and Odontology, University of Valencia, Av. de Blasco Ibáñez, 15. 46010. Valencia, Spain; 3 Instituto de Medicina Legal y Ciencias Forenses, Calle Ricardo Muñoz Suay, s/n. 46013 Valencia, Spain; 4 INCLIVA Biomedical Research Institute, Av. de Blasco Ibáñez, 17. 46010. Valencia, Spain

**Keywords:** cardiac nodes, heart, humans, myocardial infarction, pigs

## Abstract

**Background and Aim:**

The sinoatrial node is responsible for the intrinsic electrical activation that in mammals leads to coordinated rhythmic contractions of the heart, from where it is distributed through the atrial tissue to the atrioventricular node. This study aimed to conduct a histological and morphometric study of the components and cells in cardiac nodes altered by myocardial infarction (MI) and compare them with normal tissues in humans and pigs.

**Materials and Methods:**

We analyzed 10 human hearts and 10 pig hearts that died from MI and compared them with 10 healthy control hearts from each species. Histological sections of 5 μm thickness were obtained using a microtome and stained with hematoxylin–eosin and Masson’s trichrome. The identification and assessment of the percentage of connective tissue and cellularity in the cardiac nodes were performed.

**Results:**

We observed a decreased size of cardiac nodes in humans and pigs, as well as an increased percentage of fibrosis inside the nodes, and changes in the size of the nodal cells and surrounding cardiomyocytes (decrease or hypertrophy) were observed. Cartilaginous metaplasia was also found in the cardiac skeleton of all pig samples.

**Conclusion:**

In the present study, a significant increase in collagen fibers and a decrease in cellularity were found in cardiac nodes in samples from humans and pigs with MI. These findings would explain the presence of arrhythmias, which often lead to death.

## Introduction

Through its pacemaker function, the sinoatrial node (SAN) is responsible for the intrinsic electrical activation that in mammals leads to coordinated rhythmic contractions of the heart [1, 2]. The heartbeat begins through the P cells (pacemaker cells), which generate electrical impulses that are transmitted to transitional cells located, especially in the periphery of the node. From here, these cells are responsible for transmitting the impulse to the atrial cardiomyocytes, from which it is then distributed through the atrial tissue through specialized pathways of the currently unknown organization to conduct the electrical impulse to the atrioventricular node (AVN) [3–5]. Electrical conduction is slower in the AVN than in the atrial myocardium because of the physiological pause and different electrical properties of the node, such as different expressions of ion channels like connexins [6]. SAN disease in humans and other animal species was treated with different pharmacological therapies and through pacemakers with an inconsistent implantation success rate [7–9]. Findings have generally been obtained using animal models, such as dogs, rabbits, cats, mice, and pigs [10]. Malfunction of the SAN can cause complex and fatal cardiac arrhythmias [11, 12], which can lead to heart diseases such as atrial fibrillation and heart failure, often resulting in syncope and sudden cardiac death [13, 14]. The characteristic signs of SAN malfunction include persistent bradycardia, brief or sustained sinus arrest, and bradycardia-tachycardia syndrome [15, 16], which can be observed during the acute phase of myocardial infarction (MI) in humans [17, 18]. Collagen networks in the SAN can provide structural support to nodal cells, blood vessels, nerve fibers, and other types of supporting cells to stably couple all components of the node. This collagen may also provide mechanical protection to pacemaker cells against excessive stretching caused by contraction of the surrounding myocardium [19]. Healthy human SAN is composed of 35%–55% fibrotic content, which is possibly evidenced by a lower heart rate in humans compared with other animal species [20]. However, it has been shown that fibrosis within the SAN and conduction pathways increases after heart failure and MI [19, 21]. This pathological increase in fibrosis disrupts the compressed coupling between conduction cells and impairs their function. The loss of this vital function for the compact structural support of the SAN often results in bradycardia, conduction blocks, and reentry [19].

The prevalence and severity of SAN malfunction highlight the need to conduct more detailed studies that identify and describe the structural and functional aspects of SAN, addressing them efficiently to treat and/or prevent the worsening of this disease. However, despite significant advances in our understanding of SAN functioning, the three-dimensional (3D) complexity of SAN has not been fully described [22–24]. Lesions in cardiac nodes after MI are understudied in humans and pigs. The latter species is the main animal model for cardiological studies. Studies in this regard are almost exclusively limited to the ventricular component of the conduction system.

This study aimed to perform a comparative histological and morphometric study of the fibrous and cellular components in cardiac nodes altered by MI and compare them with the structure of normal nodes in humans and pigs.

## Materials and Methods

### Ethical approval

The procedures were in accordance with the Research Ethics Committee of the Industrial University of Santander (N° 013-2023) and comply with resolution 008430 of 1993, decree 2164 of 1992, and Law 10 of 1990 of the local Ministry of Health and with the principles of the Declaration of Helsinki. In addition, they comply with Law 84 of 1989 in the national context, which corresponds to the “National Statute for the Protection of Animals,” in Chapter VI of the use of animals in experiments and research.

### Study period and location

This study was conducted from November 2022 to November 2023 in the city of Bucaramanga, Colombia.

### Processing of samples and tissue collection

Human hearts were obtained from 10 adult males (20–60 years, weight 60–80 kg) who died from MI. The hearts were obtained from necropsies carried out by the Department of Pathology of the Industrial University of Santander in Bucaramanga, Colombia, and the Institute of Legal Medicine and Forensic Sciences of the city of Valencia, Spain. Male specimens were evaluated due to the high number of individuals in this genus who suffer from infarction, excluding female specimens were excluded to avoid statistically inappropriate comparisons. Similarly, the hearts of 10 male pigs (weighing 85–90 kg and with an average age of 5 months) who experienced sudden death were obtained from necropsies performed by veterinarians. Males were also chosen because they are subjected to stress factors during transport to slaughter plants and suffer MIs, whereas in females, the incidence of MI is considerably lower because females remain with few stress factors on the farms. Ten normal human and pig hearts were evaluated as control groups.

### SAN evaluation

The SAN region was cut into 5-mm-thick slices between the junction of the superior vena cava (cranial vena cava in animals) and the right atrium in humans and pigs. The SAN is located below the sulcus terminalis and is characterized by a small epicardial ridge (crista terminalis).

### AVN evaluation

The AVN was cut serially by removing a block at the inter-atrial and interventricular septa junctions. The node was found subendocardially above the junction of the septal leaflet of the tricuspid valve and a few millimeters anterior to the orifice of the coronary sinus. The tissue was sectioned perpendicular to the line of union of the septum to obtain 6–8 samples per node.

Samples were labeled with numbers, fixed in 5% formaldehyde, and embedded in paraffin. Finally, the tissue samples were divided into 5 μm sections through a microtome and stained with hematoxylin–eosin and Masson’s trichrome. The identification and assessment of the percentage of connective tissue and cellularity in the cardiac nodes were performed.

### Image review and histopathological assessment

Samples were collected from the nodes and evaluated using a Leica DMD108 optical microscope (Leica Microsystems, Wetzlar, Germany). For morphometric evaluation, 310 SAN and AVN tissue micrographs were taken at different magnifications. The area, average density, maximum and minimum diameters, perimeter, roundness, length, and width of the nodes, as well as the node parameters, were measured at a magnification of 4×. A 4×, whereas 10× or 20× magnification was used to measure the cell parameters.

### Evaluation of cellular morphometry

In each node’s P- and T-cells and surrounding cardiomyocytes, we determined maximum and minimum diameters, mean diameter, roundness, and area (50 cells were evaluated for each cell type). To visualize the desmin intermediate filaments (IF) in the nodal cells, immunohistochemical staining was performed using anti-human desmin clone D33-IR606 (Dako Corporation. Santa Clara, United States). The visualization of nodal cell filaments was compared with that of surrounding cardiomyocytes. A computerized morphometric study was conducted using Image-Pro Plus 7.1 software (Media Cybernetics, Silver Spring, MD, USA). Through segmentation, we determined the percentage of tissue components of each node (calculated using the morphometric program), where red indicated the percentage of collagen fibers, green the percentage of cells, and yellow the percentage of fundamental substance.

### Statistical analysis

Hypothesis tests, graphical representations, and descriptive statistics were performed using SPSS 20.0 software (SPSS, Chicago, IL, USA) and Microsoft Excel 2013 (Microsoft Office, Washington, USA). Statistical significance was considered at a value of p < 0.05, and for continuous variables, a 95% confidence interval. Descriptive statistics were calculated for each morphometric parameter, and the Kolmogorov–Smirnov normality test was performed. The unpaired Student’s t-test was used for normal variables, such as node parameters, and the Mann–Whitney U test was used for quantitative variables with non-normal distribution and comparison of cells between the two species. Data are expressed as mean standard deviation (SD) for all measured lengths.

## Results

### SAN

**Figure-1:**
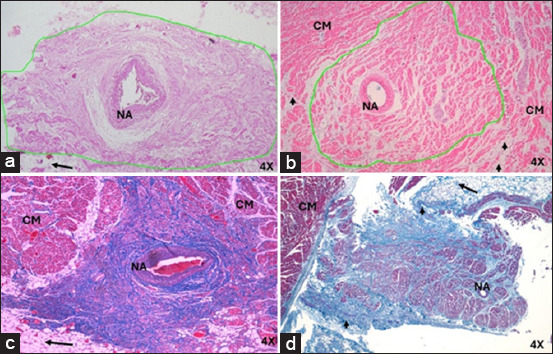
Histology of cardiac nodes in humans and pigs with acute myocardial infarction at 4×. Sinoatrial node in (a) humans and (b) pigs with hematoxylin-eosin. Sinoatrial node in (c) humans and (d) pigs with Masson’s trichrome. The infiltration of fatty tissue (arrows) at the periphery of the node in humans and pigs, which replaces the cardiomyocytes. A slight fibrotic tissue on the periphery of the node was observed in pigs (arrowheads). CM=Cardiomyocytes, NA=Nodal artery.

The SAN in humans and pigs was found in its usual location, at the juncture of the superior vena cava (cranial in animals), with the right atrium at the subendocardial level, below the terminal sulcus. The irrigating arterial branch of the SAN was located in the center of the node in both species (Figure-1).

In infarcted humans, the SAN had a rounded shape, whereas in infarcted pigs, the shape was ovoid (Figure-1); in either species, no connective tissue capsule was present in the periphery. The length and width in humans were 3.3 ± 1 mm and 1.5 ± 0.5 mm, respectively; in pigs, the node length and width were 2.6 ± 0.07 mm and 1.6 ± 0.4 mm. The morphometric parameters measured for the SAN are listed in Table-1 [5].

**Table-1:** Summary of parameters measured in the sinoatrial and atrioventricular nodes in humans and pigs with infarction compared with reported normal values.

Parameter	Infarcted humans (μm ± SD)	Infarcted pigs (μm ± SD)	p-value	Normal humans (μm ± SD)[5]	Normal pigs (μm ± SD) [5]
Sinoatrial node					
Area	3914907.75 ± 1313561.97	42899902 ± 1005755.37	0.439	1584872.5 ± 996466.4	1525082.5 ± 756809.5
Mean density	0.21 ± 0.04	0.15 ± 0.02	0.121	0.24 ± 0.06	0.30 ± 0.12
Max. diameter	3294.43 ± 662.53	2878.92 ± 203.22	1.000	2017.5 ± 517.6	2058.8 ± 586.5
Min. diameter	1473.12 ± 432.22	1802.32 ± 388.62	0.439	915.6 ± 398.4	827 ± 169.3
Perimeter	8578.41 ± 1510.03	8154.75 ± 972.90	1.000	45301.8 ± 1416.9	5228.7 ± 1398
Roundness	1.55 ± 0.36	1.24 ± 0.003	0.121	1.81 ± 0.94	1.47 ± 0.13
Atrioventricular node					
Area	750535.1 ± 122987.69	907245 ± 338339.52	0.406	1617321 ± 381970.1	1541833.3 ± 443166.8
Mean density	0.17 ± 0.004	0.17 ± 0.02	0.882	0.23 ± 0.03	0.24 ± 0.04
Max. diameter	1871.89 ± 215.62	1860.89 ± 347.79	0.960	2227.5 ± 404.7	2080.3 ± 410.6
Min. diameter	425.83 ± 109.78	596.17 ± 177.63	0.150	915.9 ± 272-4	916.4 ± 173.9
Perimeter	4597.92 ± 486.70	4472.51 ± 781.89	0.801	4604.35 ± 3226.64	5449.30 ± 852.51
Roundness	2.32 ± 0.39	1.86 ± 0.53	0.199	1.98 ± 0.61	1.64 ± 0.29

Areas were measured in μm^2^. Roundness has no units of measure. SD=Standard deviation

**Figure-2:**
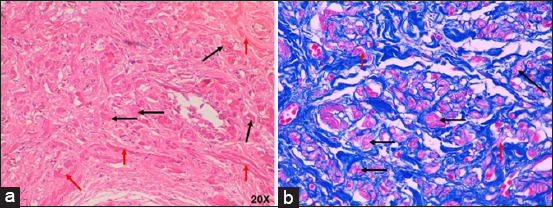
Sinoatrial node from infarcted humans stained with (a) hematoxylin-eosin at 20× and (b) Masson’s trichrome at 40×. P-cells are indicated by black, and T-cells are indicated by red arrows. CM=Cardiomyocytes.

**Figure-3:**
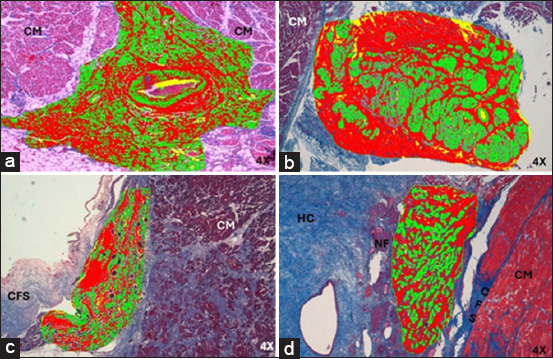
Morphometric analysis of SAN in (a) humans and (b) pigs and AVN in (c) humans and (d) pigs infarcted at 40×. The large number of collagen fibers inside the heart nodes in both species represented by red and the decrease in cells represented by green. Yellow indicates the fundamental substance of the heart nodes. CM=Cardiomyocytes, CFS=Cardiac fibrous skeleton, NF=Nerve fibers, HC=Hyaline cartilage, SAN=Sinoatrial node, AVN=Atrioventricular node.

In infarcted humans, changes were observed in SAN cells and their structural components; in P cells, we observed hypertrophy, and in T cells, a decrease in size compared with control cases (Table-2 and Figure-2). The nodal cells were easily observed because of their pale color, and the structure of the node was identified. At the periphery of the node, we observed a large amount of adipose tissue replacing the surrounding cardiomyocytes (Figure-1). Another important finding was the increased percentage of collagen fibers (fibrosis) and decreased percentage of cells inside the SAN compared to control cases (Table-3 and Figure-3) [25].

**Table-2:** Overall summary of morphometric parameters of P-cells, T-cells, and cardiomyocytes in infarcts from the sinoatrial and atrioventricular nodes in humans and pigs compared with reported normal values.

Species	Cell	Node	Area (μm ± SD)		Max. diameter (μm ± SD)		Min. diameter (μm ± SD)		Mean diameter (μm ± SD)		Roundness (μm ± SD)	
				
			I	N	I	N	I	N	I	N	I	N
Human	P cell	SAN	170.85 ± 61.69	57.9 ± 30.3	16.62 ± 3.50	9.9 ± 2.5	12.04 ± 2.18	6.3± 1.8	14.22 ± 2.61	8.1 ± 1.9	1.12 ± 0.04	1.14 ± 0.2
		AVN	101.42 ± 26.70	17.8 ± 5.3	12.77 ± 1.63	5.2 ± 0.9	9.07 ± 1.91	3.7 ± 0.7	10.95 ± 1.33	4.4 ± 0.7	1.09 ± 0.14	1.03 ± 0.05,
Pig		SAN	147.86 ± 57.30	49.7 ± 14.8	18.20 ± 7.15	8.9 ± 1.5	10.12 ± 2.49	6.4 ± 1.1	13.47 ± 2.37	7.5 ± 1.1	1.69 ± 2.29	1.07 ± 0.04
		AVN	250.72 ± 114.65	377.4 ± 151	20.58 ± 5.22	5.2 ± 0.9	14.33 ± 3.19	17.6 ± 3	17.11 ± 3.66	21 ± 4	1.17 ± 0.08	1.16 ± 0.07
Human	T cell	SAN	297.17 ± 162.15	825. ± 423	40.26 ± 17.44	69.6 ± 22.4	7.80 ± 1.93	13.8 ± 5	18.52 ± 4.32	34.5 ± 8.2	2.46 ± 0.97	3.1 ± 1.2
		AVN	138.32 ± 36.44	573. ± 323.7	19.67 ± 2.67	59.7 ± 26.7	8.04 ± 1.58	10.9 ± 3.2	13.12 ± 1.46	28.2 ± 7.1	1.37 ± 0.14	2.85 ± 1.19
Pig		SAN	310.37 ± 128.97	508 ± 463.2	43.88 ± 12.02 compared to	49.1 ± 21.8	7.55 ± 2.79	11.1 ± 4.9	15.43 ± 18.89	25.8 ± 10	2.77 ± 1.05	2.3 ± 0.6
		AVN	808 ± 424.31	715.2 ± 389	67.21 ± 19.23	63.7 ± 24.8	12.22 ± 3.55	13.1 ± 3.5	29.54 ± 11.18	31.4 ± 7.2	2.81 ± 0.77	2.7 ± 1.21
Human	Cardiomyocyte	SAN	125.09 ± 32.97	77.4 ± 22.6	13.34 ± 1.80	11.1 ± 1.7	10.62 ± 1.49	8.1 ± 1.5	12.01 ± 1.59	9.5 ± 1.4	1.05 ± 0.02	1.09 ± 0.03
		AVN	115.66 ± 30.17	70.8 ± 41.5	13.73 ± 3.27	10.3 ± 3.5	9.62 ± 1.67	7.4 ± 2.1	11.70 ± 1.75	8.8 ± 2.7	1.09 ± 0.17	1.08 ± 0.05,
Pig		SAN	226.18 ± 49.25	101.6 ± 27	21.55 ± 10.02	12.5 ± 1.7	13.68 ± 2.69	9.5 ± 1.4	16.57 ± 2.15	10.9 ± 1.4	1.37 ± 1.00	1.09 ± 0.02
		AVN	274.19 ± 69.75	100.5 ± 24.7	21.37 ± 3.60	13.1 ± 2	15.01 ± 2.43	9.1 ± 2	17.86 ± 2.38	10.8 ± 1.4	1.11 ± 0.04	1.11 ± 0.06
Scheme			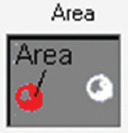		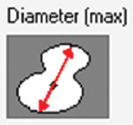		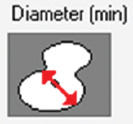		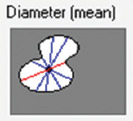		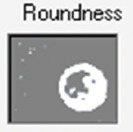	

Areas were measured in μm^2^ . Roundness has no units of measure. I=Infarcted. N=Normal [5]. SD=Standard deviation

**Table-3:** Structural components of cardiac nodes expressed as percentages in infarcted and normal human and pig hearts.

Species	Humans				Pigs			
		
Node	Infarcted SAN	Infarcted AVN	Normal SAN	Normal AVN [25]	Infarcted SAN	Infarcted AVN	Normal SAN	Normal AVN [25]
% Collagen fibers	58	52.3	52.6	38.9	63.4	77.7	45	67.5
% Fundamental substance	6	12.6	2.3	15.5	4.9	2.4	10.6	12.5
% Cells	36	35.1	45.1	45.6	31.7	19.9	44.4	20

SAN=Sinoatrial node, AVN=Atrioventricular node

**Figure-4:**
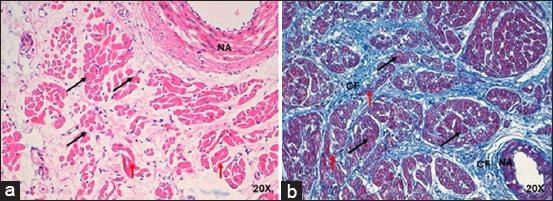
(a) Sinoatrial node from infarcted pigs stained with hematoxylin-eosin and (b) Masson’s trichrome at 20×. P-cells are indicated by black, and T-cells are indicated by red arrows. CF=Collagen fibers, NA=Nodal artery.

In infarcted pigs, the shape of the SAN was ovoid (Figure-1), P cells were hypertrophic, and a slight decrease in size was observed in T cells compared to control cases (Table-2 and Figure-4). The identification of cellular components was facilitated because the cells were paler than the surrounding cardiomyocytes. A slight fibrotic tissue was observed on the periphery of the node (Figure-1). We observed a slight reduction in the percentage of cells and a slight increase in collagen fibers (fibrosis) inside the SAN compared to control cases (Table-3 and Figure-3) [25].

No statistically significant differences were observed between the morphometric parameters measured in the SAN of humans and pigs (area, minimum diameter, width p = 0.439; density mean, roundness p = 0.121; maximum diameter, perimeter, length p = 1.000). Most parameters measured in nodal cells did not differ significantly between humans and pigs. Nonetheless, the minimum diameter of P cells was greater in humans than in pigs (p = 0.007), and the area and diameters of surrounding cardiomyocytes were significantly larger in pigs than in humans (p < 0.001).

### AVN

**Figure-5:**
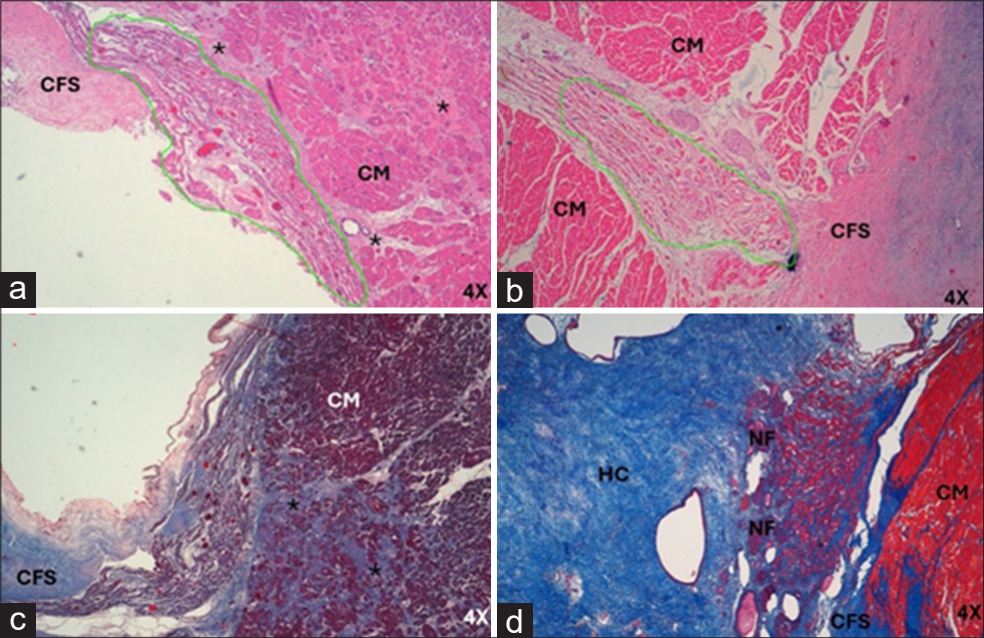
(a) Histology of infarcted atrioventricular node from humans and (b) pigs stained with hematoxylin-eosin. (c) Atrioventricular node in humans and (d) pigs stained with Masson’s trichrome at 4×. CM=Cardiomyocytes, CFS=Cardiac fibrous skeleton, HC=Hyaline cartilage, NF=Nerve fibers, (*)=Fibrosis zone.

In infarcted humans and pigs, the AVN was located subendocardially near the exit orifice of the coronary sinus and in front of the septal leaflet of the tricuspid valve in the right half of the interatrial septum. The arterial branch that irrigates the node was not present in either species. In 100% of the pigs, cartilaginous metaplasia (hyaline or fibrous cartilage) was observed near the AVN in the cardiac fibrous skeleton (Figure-5). In humans and infarcted pigs, the AVN showed an ovoid shape (Figure-5). The length and width of the node in humans were 1.9 ± 0.2 mm and 0.5 ± 0.1 mm, respectively, while in pigs, it was 1.7 ± 0.3 mm and 0.6 ± 0.2 mm. The morphometric parameters measured at the node are presented in Table-1 [5].

**Figure-6:**
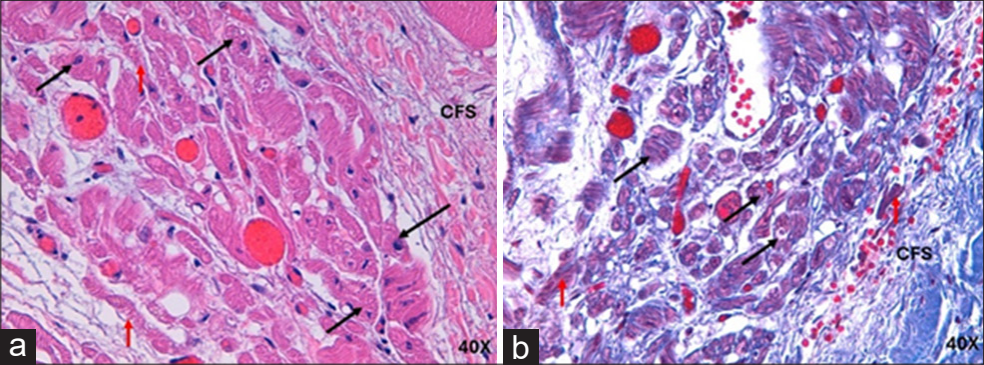
(a) Atrioventricular node from infarcted humans stained with hematoxylin-eosin and (b) Masson’s trichrome at 40×. The P-cells are indicated by black and T-cells are indicated by red arrows. CFS=Cardiac fibrous skeleton.

Compared with the control group, the AVN of infarcted humans showed a decrease in the cell package percentage and an increased percentage of collagen fibers (Table-3 and Figure-3) [25]. Nodal cells were paler than surrounding cardiomyocytes. In addition, P-cells were smaller than cardiomyocytes, facilitating their identification, and some of these cells presented with obvious nuclei. We observed a decrease in the size of P- and T-cells and cardiomyocytes with hypertrophy compared to control cases (Table-2 and Figure-6).

**Figure-7:**
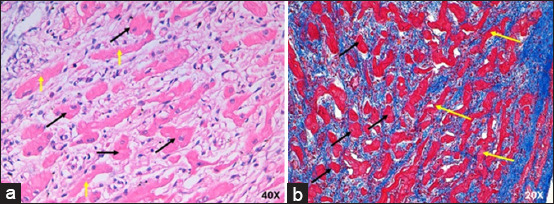
(a) Atrioventricular node of infarcted pigs stained with hematoxylin-eosin at 40× and (b) Masson’s trichrome at 20×. P-cells are indicated by black, and T-cells are indicated by yellow arrows.

In infarcted pigs, the percentage of collagen fibers increased, and the cell percentage was the same as in control cases (Table-3 and Figure-3) [25]. The AVN was easily identified owing to the large size and paler color of the nodal cells. Compared with the control, P cells were substantially decreased in size, T cells showed mild hypertrophy, and the surrounding cardiomyocytes were slightly decreased in size (Table-2 and Figure-7).

No statistically significant differences between humans and pigs were observed in the morphometric parameters measured in the AVN (area p = 0.406; density mean p = 0.882; maximum diameter p = 0.960; minimum diameter p = 0.150; perimeter p = 0.801; roundness p = 0.159; length p = 0.578; and width p = 0.427). However, the area, diameter, and roundness of P-cells, T-cells, and cardiomyocytes in the AVN were significantly greater in pigs than in humans (p < 0.001).

### Immunohistochemistry

**Figure-8:**
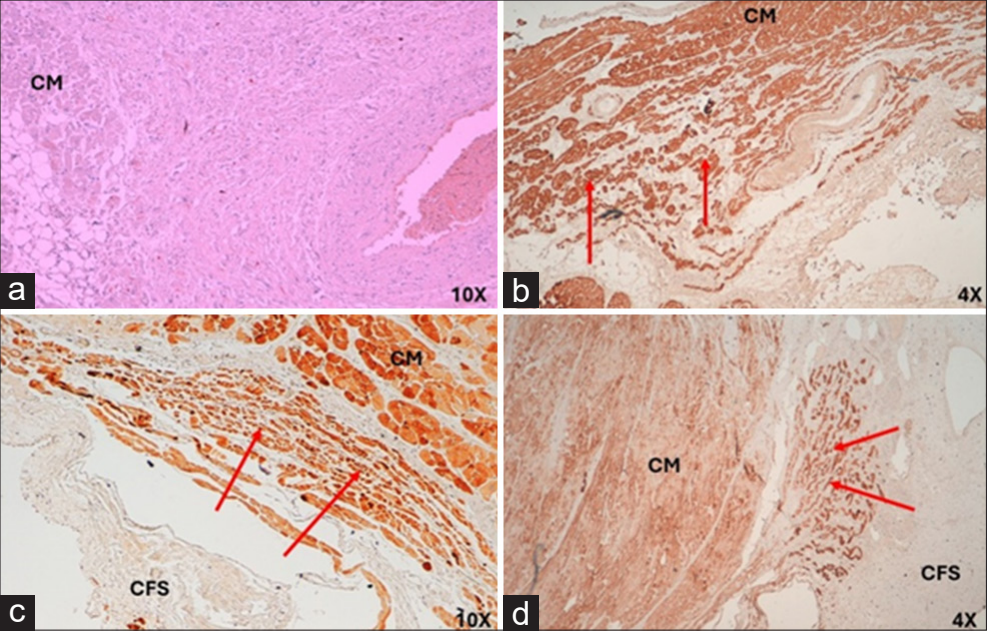
(a) Identification of SAN cells in humans and (b) pigs and (c) AVN cells in humans and (d) pigs by desmin immunohistochemical staining in infarcted animals at 4× and 10×. The difficulty in distinguishing nodal cells from cardiomyocytes in hearts that have suffered myocardial infarction from both species in SAN and from humans in AVN, observing a higher intensity difference between nodal cells (arrows) and CM. CFS=Cardiac fibrous skeleton, SAN=Sinoatrial node, AVN=Atrioventricular node, CM=Cardiomyocytes.

We used desmin staining to improve the identification of nodal cells. In humans, SAN exhibited a negative reaction to immunohistochemistry in both nodal cells and surrounding cardiomyocytes. In the AVN, a strong positive reaction was observed in both nodal cells and cardiomyocytes, which precludes the use of this test as an identification protocol for infarctions. The test showed high positivity for SAN cells and cardiomyocytes in pigs without possibly identifying the structures. In the AVN, there was greater positivity in nodal cells than in cardiomyocytes, allowing easy identification of the structures in cases of infarction (Figure-8).

## Discussion

Under normal conditions, the SAN in humans has been described as having an ovoid shape, but its average length has been the subject of debate, mainly because of early anatomical without precise histological descriptions, which reported measurements of between 10 and 15 mm, whereas more recent morphometric studies have found shorter lengths of between 3 and 4 mm [5, 26–29]. In our study of infarctions, we observed variations in the SAN, such as since it has a rounded shape and shorter length than that reported in normal conditions (3.3 mm), this can generate supraventricular arrhythmias, which alter the conduction of the electrical impulse.

In humans, under normal conditions, the fibrotic content of SAN is between 35% and 55%, and it provides mechanical protection to the pacemaker cells and other components within the node [20]. In MI, this fibrotic tissue has been shown to increase and interrupt coupling between conduction cells, which alters their function and leads to bradycardia, conduction blocks, and re-entry [19]. In our study in infarcted humans, in accordance with previous studies [19, 20], we have been able to demonstrate an increased percentage of collagen fibers (fibrosis) and a marked decrease in the percentage of conduction cells, which would increase the possibility of alterations in cardiac conduction.

The main conduction cells (P cells) have a reported diameter of 3–10 μm under normal conditions in humans [5, 28, 29]. That hypertrophic P cells were found in MI in our study indicates that these cells seek a compensatory mechanism to try to transmit the electrical impulse in the most appropriate way, and alongside the increased fibrosis and decreased number of cells present within the SAN, this further complicates the correct transmission of cardiac impulses. We also observed a decrease in the size of T cells, which support pacemaker cells for impulse transmission to cardiomyocytes, significantly hindering their continuity.

The ovoid shape of the SAN found in our study in infarcted pigs is consistent with the shape of the node under normal conditions [5]. In contrast, the reported length of this structure (4–18 mm) in pigs without a history of clinical heart disease [5, 30] is greater than that observed in our study under MI conditions, demonstrating that this pathological condition can affect the functionality of the SAN by decreasing its size.

Similar to our findings in humans, in infarcted pigs, we observed a higher percentage of collagen fibers (fibrosis) inside the SAN and a lower cellular content percentage, indicating that these alterations in the basic structure of the node can lead to poor conduction, which may result in arrhythmia development.

Previous studies by Gomez-Torres *et al*. [5] and Opthof *et al*. [30] indicated that the size of pacemaker cells (P-cells) in pigs under normal conditions is between 4 μm and 9 μm and that T-cells are 49.1 μm [5], but in our study in infarcted pigs, we found marked hypertrophy of P-cells and a slight decrease in T-cell size. This demonstrates that pacemaker cells increase their size to implement a compensatory mechanism to adequately transmit the electrical impulse due to the low cell population observed post-infarction and the increase in fibrosis within the SAN that further complicates electrical transmission. This parallel finding in humans indicates a similarity between two species that persists under similar pathological conditions.

Under normal conditions, the human AVN has an ovoid shape and a length of 2.2–7 mm [5, 31, 32]. Our MI study found the AVN size slightly smaller than that indicated as a lower threshold in previous studies [5, 31, 32]. An analysis of the components of the AVN in infarcted humans revealed that the fibrosis percentage showed a marked increase and the percentage of cells inside the node decreased consistently compared with normal human hearts (collagen fibers = 38.9%; cells = 45.6%) [25].

One of the main identifying characteristics of the human AVN is the smaller P-cells than cardiomyocytes. In our study of MI, we observed that the size of these cells, like that of T-cells, decreased further, and the cardiomyocytes were hypertrophied as a compensatory method in MI. This comparison was based on the size described in previous studies of normal hearts, in which P cells were 5–18 μm [5, 33]. T-cells were 59.7 μm and cardiomyocytes were 23.1 μm [5]. This damage to atrioventricular conduction reduces the impulse transmission between the atria and ventricles, thereby generating ventricular arrhythmias that can be fatal.

In the evaluation of pigs under normal conditions, it was observed that the AVN had an ovoid shape and length of 2 mm [5]. In our study of infarcts, the length was slightly shorter, and the ovoid shape observed in healthy hearts was retained. When evaluating the tissue components inside the node in our study of infarcted pigs, we observed that the percentage of collagen fibers increased considerably, and the percentage of cells decreased slightly compared with the hearts of non-infarcted pigs, in which fibrosis was 65.7% and the cellular component was 20% [25]. Interestingly, all the samples in our study exhibited cartilaginous metaplasia in the cardiac skeleton, which, in theory, could increase the level of damage in the atrioventricular zone. However, cases of cartilage in this area without pre-existing heart disease [25] were very similar to our results in this study.

In the non-infarcted hearts of pigs, the P-cells of the AVN have a reported diameter of 45.3 μm, T-cells 63 μm, and the surrounding cardiomyocytes 27.5 μm [5]. Comparing them with the diameters found in our study in infarcted pigs, we observed a decreased size of P-cells and cardiomyocytes. However, T-cells displayed a size similar to that of the aforementioned report. This shows that altered electrical conduction also occurs in the AVN, which can compromise the correct transmission of this impulse and lead to arrhythmias in the ventral region of the heart.

Desmin IF is the main component of the cytoskeleton of nodal cells, and it is present in greater quantities in the cells of the cardiac conduction system than in cardiomyocytes because desmin is located throughout the entire cytoplasm, not only at specific points like in cardiac muscle cells [34, 35]. This immunohistochemical test in the nodal cells of humans and pigs without MI has proven effective for cell identification to distinguish them from the cardiomyocytes surrounding the cardiac nodes [5]. In our study of infarcts, this test was impossible to apply to the SAN of either humans or pigs because we observed an alteration in the positivity of desmin, which precluded full identification of the cells. In the AVN, the test in humans also showed alteration by finding high positivity in nodal cells and cardiomyocytes without allowing cell identification; nonetheless, it was possible to use desmin as a criterion for identifying AVN cells in infarcted pigs since it showed high positivity compared to cardiomyocytes.

The strength of the present histomorphometric study was in its evaluation of factors that were reported qualitatively in previous studies [19, 20], such as the assessment of collagen fiber percentage, cell number, and size in cardiac nodes in normal specimens and infarction. The weakness of this study is the impossibility of contrasting these findings between specimens of both sexes in humans and pigs because of the very low incidence of infarction in females.

## Conclusion

Cardiac alterations leading to MI change the size, tissue structure, and diameter of cells inside the cardiac nodes in humans and pigs, predisposing to the presence of supra- or ventricular cardiac arrhythmias that impair the quality of life of patients who survive or cause irreversible damage, leading to sudden cardiac death. The use of immunohistochemical staining, such as desmin, in the presence of MI is not an efficient method for identifying nodal cells. This study focused on the changes that occur in the cardiac nodes after a myocardial infarction, which is relevant information for preventing or treating cardiac arrhythmias that may develop after an ischemic event. In the future, research should focus on determining at the molecular level what type of genes or proteins are responsible for the development of atheromatous plaques, which lead to poor irrigation of the components of the cardiac conduction system and therefore to the development of arrhythmias.

## Authors’ Contributions

FGT, LEBA, PMA, CRN, and ARS: Study conception and design, material preparation and data collection and analysis. FGT: Drafted and revised the manuscript. All authors have read and approved the final manuscript.
